# Phytochemical and Cytotoxic Investigations of *Curcuma mangga* Rhizomes

**DOI:** 10.3390/molecules16064539

**Published:** 2011-05-31

**Authors:** Sri Nurestri A. Malek, Guan Serm Lee, Sok Lai Hong, Hashim Yaacob, Norhanom Abdul Wahab, Jean-Frédéric Faizal Weber, Syed Adnan Ali Shah

**Affiliations:** 1 Institute of Biological Sciences, University of Malaysia, 50603 Kuala Lumpur, Malaysia; E-Mails: leeguanserm@hotmail.com (G.S.L.); chrishsl@hotmail.com (S.L.H.); 2 International University College of Nursing, L1.10, Cova Square, Jalan Teknologi, Kota Damansara, PJU5, 47810 Petaling Jaya, Selangor Darul Ehsan, Malaysia; E-Mail: imya12@yahoo.com; 3 Centre for Foundation Studies in Science, University of Malaysia, 50603 Kuala Lumpur, Malaysia; E-Mail: norhanom@um.edu.my; 4 Research Institute of Natural Products for Drug Discovery (RiND), Faculty of Pharmacy, Universiti Teknologi MARA (UiTM), Puncak Alam Campus, 42300 Bandar Puncak Alam, Selangor Darul Ehsan, Malaysia; E-Mails: jffweber@salam.uitm.edu.my (J.-F.F.W.); syedadnan@salam.uitm.edu.my (S.A.A.S.)

**Keywords:** *Curcuma mangga*, Zingiberaceae, labdane diterpene, neutral red cytotoxicity assay, cancer cell lines

## Abstract

Investigations on the cytotoxic effects of the crude methanol and fractionated extracts (hexane, ethyl acetate) *C. mangga* against six human cancer cell lines, namely the hormone-dependent breast cell line (MCF-7), nasopharyngeal epidermoid cell line (KB), lung cell line (A549), cervical cell line (Ca Ski), colon cell lines (HCT 116 and HT-29), and one non-cancer human fibroblast cell line (MRC-5) were conducted using an *in-vitro* neutral red cytotoxicity assay. The crude methanol and fractionated extracts (hexane and ethyl acetate) displayed good cytotoxic effects against MCF-7, KB, A549, Ca Ski and HT-29 cell lines, but exerted no damage on the MRC-5 line. Chemical investigation from the hexane and ethyl acetate fractions resulted in the isolation of seven pure compounds, namely (*E*)-labda-8(17),12-dien-15,16-dial (**1**), (*E*)-15,16-bisnor-labda-8(17),11-dien-13-on (**2**), zerumin A (**3**), β-sitosterol, curcumin, demethoxycurcumin and bis-demethoxycurcumin. Compounds **1** and **3** exhibited high cytotoxic effects against all six selected cancer cell lines, while compounds **2** showed no anti-proliferative activity on the tested cell lines. Compound **1** also demonstrated strong cytotoxicity against the normal cell line MRC-5. This paper reports for the first time the cytotoxic activities of *C. mangga* extracts on KB, A549, Ca Ski, HT-29 and MRC-5, and the occurrence of compound **2** and **3** in *C. mangga*.

## 1. Introduction

Cancer is one of the leading causes of deaths in Malaysia. It is the fourth leading cause of death after diseases of the circulatory and respiratory systems and motor vehicle accidents, and responsible for 10.3% of medically certified deaths. In 2002, about 26,089 cases of cancer were diagnosed in Peninsular Malaysia and an estimated 10,656 cases were not reported. As many as 30,000 new cases are reported yearly [1,2,3]. Breast cancer is the most common cancer in in women of all ethnic groups aged 20 and above, while lung cancer is the most common cancer among males of all the three major ethnic groups [2,3]. In the United States, death due to cancer is second only to heart disease. Although many anti-cancer drugs used clinically today are very effective in killing cancer cells, they however have undesirable side effects like excessive hair loss, nausea and loss of appetite. It is widely known that many drugs used today are derived from natural products. A popular example is taxol, derived from the Pacific yew tree, which is effective against breast, ovarian and cervical cancers. 

Natural products provide a wide variety of phytochemicals which have been shown to have a broad spectrum of activity across multiple signalling pathways. Natural products not only disrupt aberrant signalling pathways leading to cancer (*i.e.*, proliferation, deregulation of apoptosis, angiogenesis, invasion and metastasis), but also synergize with chemotherapy and radiotherapy [4]. It is not surprising therefore that many researchers are constantly turning to natural products for alternative source of medicines.

*Curcuma mangga*, locally known as “temu pauh” or “kunyit mangga”, meaning mango-like turmeric, is a rhizomatous herb of the *Zingiberaceae* family. The rhizomes of *C. mangga* are used in Java as a seasoning for food and treatment for stomach aches, fever and cancer related diseases. Although there have been reports concerning the chemical constituents and biological activities of *Curcuma mangga*, only a few have focused on the cytotoxic activity against human tumour cells [9,10]. Four reports have shown that *C. mangga* possesses antioxidant, antitumour, antifungal and anti-allergic properties [5,6,7,8,10].

In the present study, we investigated the cytotoxic effects of the crude and fractionated extracts of *C. mangga* against six human cancer cell lines, namely the hormone-dependent breast cell line (MCF-7), nasopharyngeal epidermoid cell line (KB), lung cell line (A549), cervical cell line (Ca Ski), colon cell line (HCT 116), colon cell line (HT-29) and one non-cancer human fibroblast cell line (MRC-5), using an *in-vitro* neutral red cytotoxicity assay. We also identified the components in the active hexane and ethyl acetate fractions of *C. mangga* rhizomes. The chemical investigation led to the isolation of seven compounds, namely (*E*)-labda-8(17),12-dien-15,16-dial (**1**), (*E*)-15,16-bisnorlabda-8(17),11-dien-13-one (**2**), zerumin A (**3**), β-sitosterol, curcumin, demethoxycurcumin and bisdemethoxycurcumin. Compounds **2** and **3** are reported for the first time in this plant. To our knowledge, there is no report on the cytotoxic activities of *C. mangga* on the above mentioned human cancer cell lines, except for the MCF-7 and HCT 116 cell lines [9,10].

## 2. Results and Discussion

### 2.1. Cytotoxic Activities of the Crude Extracts and Fractions

The results of preliminary cytotoxic screening of the methanol and fractionated extracts of *C. mangga* are summarized in Table 1.

The methanol extract exhibited mild cytotoxic effects against all selected human cancer cell lines, with IC_50_ values ranging from 22.0 to 36.8 μg/mL. The ethyl acetate fraction only showed good activity against HT-29, with IC_50_ 18.5 μg/mL, whilst it showed mild inhibitory activity against the other investigated cell lines. The hexane fraction of *C. mangga* exhibited good activities against all the cell lines tested (except HCT 116) with IC_50_ values of 8.1, 15.4, 17.4, 11.4 and 17.9 μg/mL against the MCF-7, KB, A549, Ca Ski and HT-29 cell lines, respectively. Interestingly, the hexane fraction showed no adverse effect on the non-cancer human fibroblast cell line MRC-5 (IC_50_ >100 μg/mL). If this also occurs *in vivo*, the hexane extract would be considered safe for human consumption and its non-toxic effect on the MRC-5 cell line merits consideration for further toxicity and clinical studies. It was also observed that the crude methanol and the ethyl acetate extracts caused no damage to the normal human fibroblast cell line. The water extract did not show any cytotoxicity against any of the tested human cancer cell lines (IC_50_ >100 μg/mL). As the hexane fraction demonstrated good inhibitory activity and the ethyl acetate fraction exhibited mild activity, further chemical investigation was focussed on these two fractions. This led to the isolation of compounds **1-3**.

### 2.2. Isolation and Identification of Compounds ***1-3***

**Figure 1:**
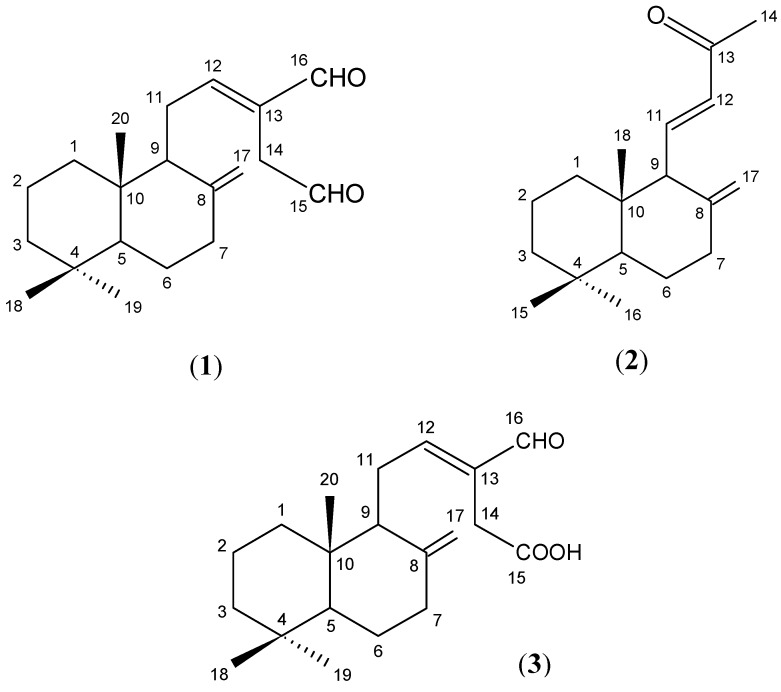
Chemical structures of compounds **1-3**.

Repeated silica gel column chromatography and HPLC separation of the hexane fraction afforded (*E*)-labda-8(17),12-dien-15,16-dial (**1**), (*E*)-15,16-bisnorlabda-8(17),11-dien-13-one (**2**) and β-sitosterol. The ethyl acetate fraction afforded zerumin A (**3**) and the curcuminoids. Compounds **2** and **3** are reported for the first time in this plant. All chemical components (Figure 1) were identified by comparison of their mass and NMR spectra with published data and found to be in good agreement [13,14].

### 2.3. Cytotoxic Activities of the Isolated Pure Compounds

The isolated compounds were further tested on the selected cancer cell lines as the hexane fraction showed strong cytotoxic effect against MCF-7, KB, A549, Ca Ski and HT-29 cells, whilst the ethyl acetate fraction exhibited moderate effects on KB, A549, HCT 116 and HT-29 cells. Table 2 summarizes the results of the cytotoxic activity of the isolated compounds **1-3**. Compound **1** displayed remarkable cytotoxic activity, with IC_50_ values 4.3, 7.6 and 6.3 μg/mL against MCF-7, HCT 116 and HT-29 cell lines, respectively. Compound **2** exhibited no cytotoxic activity on any of the investigated human cancer cell lines. In addition, compound **3** in the ethyl acetate fraction demonstrated strong activity, with IC_50_ values of 8.7, 9.2 and 9.3 μg/mL against MCF-7, A549 and HCT 116 cell lines, respectively. Compound **1** and **3** are structurally related; they only differ at C-15 which in **1** carries an aldehyde group, whilst it is a carboxylic acid group in **3**. However, both compounds **1** and **3** showed cytotoxic effects on the normal fibroblast lung cell lines (IC_50_ 8.9 and 16.2 μg/mL, respectively). The cytotoxic effect shown by the hexane fraction may be due to the presence of compound **1** which comprises of 1.5% (w/w) in this fraction, whilst compound **3** and the curcuminoids may be responsible for the observed activity of the ethyl acetate fraction.

In the present study, doxorubicin, used clinically for the treatment of a great variety of cancer diseases, was the positive control [16,17,18]. However, this commercial chemotherapeutic drug not only demonstrated remarkable toxicity against all tested cancer cell lines (IC_50_ values from 0.05 to 0.58 μg/mL), but also on the human normal cell line (IC_50_ value 0.40 μg/mL). Due to its high toxicity on human normal cell lines, the continuous use of doxorubicin can cause major adverse effects to human health [16]. Even though the level of cytotoxicity of the pure compounds is not as high as doxorubicin, they however, have comparably lower toxicity against normal cells. Hence, the use of these compounds as anticancer agents in combination with other cytotoxic therapeutic drugs, may reduce the overall adverse effects of the drugs. At this stage, it is not possible to justify the use of the isolated compounds in the treatment of cancer. A more comprehensive study involving experimental animals and clinical investigation is required.

## 3. Experimental

### 3.1. General

*NMR analysis*: ^1^H- and ^13^C-NMR spectra were obtained on Bruker Avance III 500 MHz Nuclear Magnetic Resonance spectrophotometers. 

 

*GC-MS analysis*: GC-MS analysis was performed using an Agilent Technologies 6980N gas chromatograph equipped with a 5975 Mass Selective Detector (70 eV direct inlet); the HP-5ms capillary column (5% phenylmethylsiloxane) with column dimensions 30.0 m × 25 mm × 25 μm was initially set at 150 °C, then the temperature of the oven was increased at 5 °C per minute to 300 °C using helium as carrier gas at flow rate 1 mL/min. The total ion chromatogram obtained was auto integrated by ChemStation and the components were identified by comparison with the supplied mass spectral data database (NIST 05 Mass Spectral Library, USA) whenever possible.

 

*HPLC analysis*: HPLC analysis was performed on a Shimadzu LC system equipped with a Shimadzu LC-10AT VP pump, Shimadzu SCL-10A VP system controller, Shimadzu SPD-M10A VP Photo Diode Array detector, Shimadzu DGU-12A vacuum degasser and Shimadzu LC Solution software. The solvents used for HPLC were of chromatographic grade acetonitrile (J.T. Baker), methanol (J.T. Baker) and ultra-pure water (H_2_O). The column used was a Chromolith Performance RP-18e (Merck) (100.0 mm × 4.6 mm i.d.) for analytical scale HPLC and semi-preparative column was a Chromolith Performance RP-18e (100.0 mm × 10.0 mm i.d.) for preparative scale separation.

 

*In-vitro neutral red cytotoxicity assay*: All cancer cell lines were purchased from the American Type Culture Collection (ATCC, USA), medium like RPMI 1640, McCOY’S 5A, Eagle Minimum Essential Medium (EMEM) from Sigma-Aldrich, USA. The supplements or antibiotics such as foetal bovine serum, penicillin/streptomycin (100 μg/mL), amphotericin B (50 μg/mL), tripsin-EDTA (100X) were purchased from PAA Lab, Austria as well as sodium pyruvate (11 mg/mL) from Sigma-Aldrich, USA. The cells were cultured in a Shel Lab water-jacketed CO_2_ incubator with 25 cm^3^ tissue culture flasks (Nunc, Denmark), which was observed routinely under inverted microscope (Leica DMI3000 B, Germany) for any contamination and seeded into 96-well flat bottom microtiter plate (Nunc, Denmark). The extract or pure compounds were dissolved in dimethyl sulfoxide (DMSO, Molecular Biology Grade, >99.9%) from Sigma-Aldrich, USA. The 96-well microtiter plate was agitated rapidly on a microtiter plate incubator shaker (LT Biomax 500) and absorbance of eluted dye measured at 540 nm using a ELISA microplate reader (EMax microplate reader of Molecular Devices, USA). Cell lines such as MCF-7, A549, Ca Ski and HT-29 were maintained in RPMI 1640 medium, HCT 116 in McCOY’S 5A Medium, KB and MRC-5 cells in Eagle Minimum Essential Medium (EMEM). All of these media were supplemented with 10% foetal bovine serum (FBS), penicillin/streptomycin, amphotericin B and only additional sodium pyruvate in EMEM. The cells were cultured in tissue culture flask in 5% CO_2_ incubator kept at 37 °C in a humidified atmosphere and observed routinely under inverted microscope from any contaminations. Each fresh medium was replaced every 2 or 3 days until cell confluence was achieved and the cells were detached by using tripsin-EDTA. 

### 3.2. Plant Materials

The authenticated rhizomes of *C. mangga* were obtained from Yogjakarta, Indonesia in July 2006. A voucher specimen (voucher number HI 1331) was deposited in the Herbarium of the Institute of Biological Sciences, Faculty of Science, University of Malaya, 50603 Kuala Lumpur, Malaysia.

### 3.3. Extraction and Isolation of Pure Compounds

**Figure 2:**
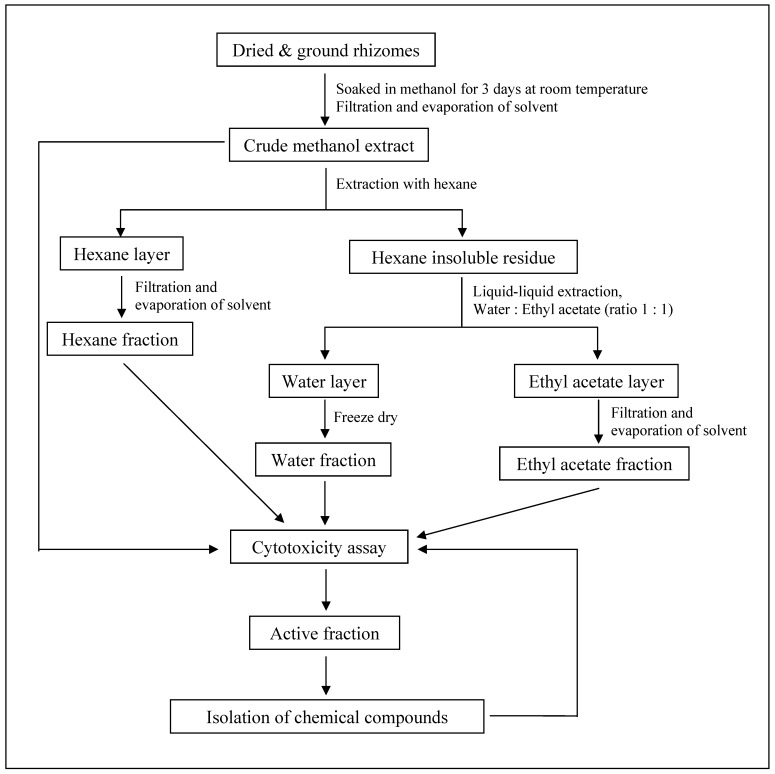
Procedures in the bioassay-guided chemical investigation of *C. mangga.*

The dried, ground rhizomes of *C. mangga* (1.0 kg) were soaked in methanol (2,000 mL) for three days at room temperature. The solvent-containing extract was then decanted, any traces of water was removed using anhydrous sodium sulphate and evaporated under reduced pressure using a rotary evaporator to give a dark brown crude methanol extract (106.4 g, 10.6%). The crude methanol extract was then extracted with hexane (500 mL) and concentrated *in vacuo* to obtain a yellowish brown hexane fraction (35.7 g, 33.6% based on crude methanol extract). The hexane insoluble residue was further partitioned using ethyl acetate and water (500 mL:500 mL) to give a dark brown ethyl acetate fraction (30.1 g, 28.3% based on crude methanol extract) and a light brown water fraction (7.4 g, 7.0% based on crude methanol extract). A summary of procedures in the bioassay-guided chemical investigation of *C. mangga* is shown in Figure 2.

The hexane fraction (1.0 g) was subjected to vacuum liquid chromatography (VLC; column 30.0 cm length × 7.0 cm id. packed with 300.0 g silica gel) initially eluting with hexane followed by hexane enriched with increasing percentages of acetone and finally with methanol. A total of nine fractions (labelled H1 to H9) were collected. Subsequently, the fractions were subjected to an analytical scale separation on the Shimadzu HPLC system. Each fraction was prepared in 1.0 mg/mL with methanol, filtered and 20 μL was injected into a Chromolith Performance RP-18e analytical column (100.0 mm × 4.6 mm i.d.) and peaks were detected by monitoring the UV absorbance at 214 nm. The mobile phase was a binary eluent of chromatographic grade acetonitrile (ACN) and ultra-pure water (H_2_O) under the following gradient conditions: 0–5 min linear gradient of eluting solvent 45 to 50% ACN; 5–11 min linear gradient of 50 to 65% ACN; 11–21 min linear gradient 65 to 100% ACN; 21–25 min isocratic using 100% ACN; 25–30 min linear gradient solvent 100 to 45% ACN at a flow rate of 1.0 mL/min. Fractions H2 (617.0 mg) and H5 (744.3 mg) were subjected to semi-preparative separation on HPLC using the same method as utilised in the analytical scale separation described above. Fraction H2 gave the following compounds: (*E*)-labda-8(17),12-dien-15,16-dial (**1**, light yellowish oil, 15.3 mg, retention time = 17.751 min) and (*E*)-15,16-bisnorlabda-8(17),11-dien-13-one (**2**, light yellowish oil, 6.0 mg, retention time = 18.416 min). β-Sitosterol (white amorphous, 10.7 mg, retention time = 14.829 min) was obtained from fraction H5.

One gram (1.0 g) of the ethyl acetate fraction was also subjected to the same VLC setup as for the hexane fraction and a total of eight fractions (labelled as E1 to E8) were collected. The fractions were then prepared and subjected to the same analytical HPLC condition as described for the hexane fraction. Two fractions, E7 (874.4 mg) and E8 (380.0 mg), were subjected to semi-preparative HPLC separation using the same method as utilised in the analytical scale separation. Fraction E7 afforded a colourless oil, zerumin A (**3**, 5.4 mg, retention time = 12.982 min). Three curcuminoids were isolated from fraction E8 using preparative thin layer chromatography technique, PTLC (developing solvent: chloroform-methanol, 95:5): curcumin (yellow amorphous solid, 5.6 mg, *R_f_* = 0.41), demethoxycurcumin (yellow amorphous solid, 3.3 mg, *R_f_* = 0.33) and bis-demethoxycurcumin (yellow amorphous solid, 3.9 mg, *R_f_* = 0.25). All pure compounds obtained were checked by TLC and HPLC to confirm their purity and then subsequently identified through their mass spectral and NMR data which were consistent with published data [13,14].

### 3.4. *In-vitro* Neutral Red Cytotoxicity Assay

The *in-vitro* cytotoxicity activity was performed using the neutral red cytotoxicity assay based on the initial protocol described by Borenfreund and Puerner, with some modifications [15,19].

## 4. Conclusions

The cytotoxic activities observed for *C. mangga* rhizomes are ascribable to the presence of the active compound **1** in the hexane fraction, and compound **3** and the curcuminoids in the ethyl acetate fraction. Even though these compounds are not as effective as doxorubicin against cancer cell lines, in comparison they have low toxicity against human normal cell lines. The present study provides important preliminary data of extracts or isolated compounds with potential anti-neoplastic properties for future work. Since *C. mangga* is widely used in traditional medicine for the treatment of cancer, the current findings provide scientific validation in the use of the rhizomes for this purpose. As the rhizomes are also widely consumed as salad in food without any known undesirable side effecat, it can be assumed that the plant is safe for consumption at the normal dose as food. *Curcuma mangga*, is therefore a promising dietary agent that holds great potential for use in chemopreventive and chemotherapeutic strategies. Nevertheless, further investigations are necessary to validate its use and to determine its mode of action.

## Figures and Tables

**Table 1:** Cytotoxic activity (IC_50_ values) of extracts and fractions of *C. mangga* against human cancer and non-cancer cell lines.

Extracts/Fractions	IC_50_ values (μg/mL)						
	MCF-7	KB	A549	Ca Ski	HCT 116	HT-29	MRC-5
Methanol	27.9 ± 0.3	24.6 ± 0.7	30.7 ± 2.0	31.5 ± 0.2	36.8 ± 3.8	22.0 ± 1.1	>100
Hexane	8.1 ± 0.2	15.4 ± 1.7	17.4 ± 0.6	11.4 ± 1.0	31.5 ± 0.1	17.9 ± 0.3	>100
Ethyl acetate	47.1 ± 0.5	23.6 ± 0.8	21.2 ± 0.7	>100	29.4 ± 0.2	18.5 ± 0.1	>100
Water	>100	>100	>100	>100	>100	>100	>100
Doxo *	0.05 ± 0.01	0.27 ± 0.01	0.58 ± 0.01	0.18 ± 0.06	0.24 ± 0.04	0.33 ± 0.03	0.40 ± 0.03

IC_50_ ≤ 20 μg/mL is generally considered to be active [11,12]; IC_50_ > 100 μg/mL is considered not active; Tabulated values are mean ± standard deviation (SD) of three replicates; Doxorubicin* was used as the reference compound.

**Table 2:** Cytotoxic activity (IC_50_ values) of isolated compounds **1-3** against human cancer and non-cancer cell lines.

Cmpds.	IC_50_ values (μg/mL)						
	MCF-7	KB	A549	Ca Ski	HCT 116	HT-29	MRC-5
**1**	4.3 ± 1.30	14.5 ± 0.87	19.9 ± 0.38	12.1 ± 0.35	7.6 ± 0.23	6.3 ± 0.26	8.9 ± 0.49
**2**	>100	>100	>100	>100	>100	>100	>100
**3**	8.7 ± 0.29	11.7 ± 1.15	9.2 ± 0.62	14.2 ± 0.06	9.3 ± 0.50	14.9 ± 0.40	16.2 ± 0.25
Doxo *	0.05 ± 0.01	0.27 ± 0.01	0.58 ± 0.01	0.18 ± 0.06	0.24 ± 0.04	0.33 ± 0.03	0.40 ± 0.03

1 = (*E*)-labda-8(17),12-dien-15,16-dial; 2 = (*E*)-15,16-bisnorlabda-8(17),11-dien-13-one; 3 = zerumin A; Tabulated values are mean ± standard deviation (SD) of three replicates; Doxorubicin* was used as the reference compound.
